# Supplementary material for: Few Fixed Variants between Trophic Specialist Pupfish Species Reveal Candidate Cis-Regulatory Alleles Underlying Rapid Craniofacial Divergence
Source: Mol Biol Evol. 2020 Sep 2;38(2):405–23. doi: 10.1093/molbev/msaa218 (PMC7826174; doi:10.1093/molbev/msaa218)
Supplement: msaa218_Supplementary_Data [file msaa218_supplementary_data.pdf]

Supplement: Few fixed variants between trophic specialist pupfish species reveal candidate *cis*-regulatory alleles underlying rapid craniofacial divergence

Joseph A. McGirr[1], Christopher H. Martin[2]

[1]Environmental Toxicology Department, University of California, Davis, CA 95616

[2]Department of Integrative Biology and Museum of Vertebrate Zoology, University of California, Berkeley, CA 94720

Running Title: *Cis*-regulatory alleles underlying craniofacial divergence

Keywords: RNAseq, F1 hybrid, trophic specialization, allele specific expression, adaptive radiation, ecological speciation

Correspondence: Christopher Martin

Email: chmartin@berkeley.edu

Word count abstract: 246; Word count main text: 7,217; Figures: 8; Tables: 1.

Table S1. Protein coding genes near 157 SNPs and 87 deletions fixed between molluscivores and scale-eaters (within 10 kb of the first or last exon).

near fixed SNP	near fixed deletion
cckar	*acat2*
cdc14ab	*acvr1c*
cdk5r1	*adra2db*
cxcr1	*cckar*
dapk2	*cep170*
derl1	*col12a1*
dysf	*ctnnb1*
eef1d	*dph5*
fev	*dync2li1*
gimap2	*eef1a1*
nabp1	*fam219a*
nat14	*fgfr2*
nsmce2	*gm11992*
polg	*gpa33*
prpf4b	*hint1*
pxk	*hlf*
pycr3	*hlh-13*
sbk2	*irf1*
sgk1	*kcnq5*
slc25a29	*lyrm7*
slc38a2	*med25*
vrtn	*mprip*
washc5	*ncl1*
wdr78	*odf3l2*
wnt7b	*pdhb*
zhx2	*pld5*
znf628	*pxk*
	rabgap1l
	sh3pxd2a
	shisa2
	slc30a7
	u2af2
	upp2
	znf865

Table S2. 1,940 genes within 20 kb of 63,542 SNPs highly differentiated SNPs (*Fst* > 0.72 (genome-wide 99th percentile) and *Dxy* > 0.0083 (genome-wide 90th percentile)) were enriched for 420 biological processes (ShinyGO; *P* < 0.01).

Enrichment FDR	Genes in list	Total genes	Functional Category
4.91E-17	312	2474	Nervous system development
1.10E-16	339	2785	Anatomical structure morphogenesis
2.84E-15	331	2763	Regulation of developmental process
1.30E-14	278	2230	Cell development
3.74E-14	223	1683	Neurogenesis
1.89E-13	491	4671	Cellular developmental process
2.62E-13	209	1575	Generation of neurons
3.27E-13	263	2138	Regulation of multicellular organismal development
4.88E-13	470	4459	Cell differentiation
7.35E-13	375	3382	Regulation of multicellular organismal process
9.05E-12	187	1412	Neuron differentiation
1.15E-10	414	3952	Regulation of signaling
3.69E-10	407	3903	Regulation of cell communication
6.74E-10	441	4319	Regulation of biological quality
9.56E-10	193	1552	Plasma membrane bounded cell projection organization
1.50E-09	155	1172	Cellular component morphogenesis
1.50E-09	230	1954	Regulation of cell differentiation
1.54E-09	154	1164	Anatomical structure formation involved in morphogenesis
1.56E-09	150	1125	Regulation of anatomical structure morphogenesis
2.34E-09	143	1062	Tube development
3.09E-09	194	1589	Cell projection organization
3.09E-09	137	1008	Neuron projection development
3.89E-09	101	667	Developmental growth
5.47E-09	143	1077	Circulatory system development
7.06E-09	388	3779	Animal organ development
7.84E-09	150	1154	Neuron development
9.53E-09	141	1067	Cell morphogenesis
2.23E-08	108	756	Cell morphogenesis involved in differentiation
2.75E-08	138	1054	Embryo development
3.92E-08	362	3529	Regulation of signal transduction
6.98E-08	184	1548	Biological adhesion
7.82E-08	128	971	Regulation of cell development
7.93E-08	183	1541	Cell adhesion
8.30E-08	98	680	Plasma membrane bounded cell projection morphogenesis
8.30E-08	126	953	Regulation of nervous system development
9.47E-08	98	682	Cell projection morphogenesis
1.16E-07	116	860	Tube morphogenesis
1.16E-07	96	666	Neuron projection morphogenesis
1.76E-07	139	1099	Animal organ morphogenesis
1.76E-07	99	701	Cell part morphogenesis
1.76E-07	171	1433	Positive regulation of developmental process
2.28E-07	281	2653	Regulation of cellular component organization
2.32E-07	79	517	Axon development
2.54E-07	111	824	Head development
2.78E-07	74	474	Axonogenesis

2.78E-07	91	632	Chordate embryonic development
2.94E-07	93	652	Embryo development ending in birth or egg hatching
3.02E-07	97	691	Regulation of cell projection organization
3.11E-07	96	682	Regulation of plasma membrane bounded cell projection organization
3.50E-07	87	598	Cell morphogenesis involved in neuron differentiation
3.54E-07	94	665	Regulation of neuron differentiation
3.85E-07	450	4662	Macromolecule modification
4.38E-07	155	1286	Negative regulation of multicellular organismal process
5.54E-07	200	1775	Ion transport
6.15E-07	357	3570	Phosphate-containing compound metabolic process
6.37E-07	212	1911	Positive regulation of multicellular organismal process
6.62E-07	359	3597	Phosphorus metabolic process
7.12E-07	375	3789	Positive regulation of metabolic process
7.32E-07	111	844	Regulation of neurogenesis
7.85E-07	129	1028	Growth
1.50E-06	94	687	Blood vessel development
1.50E-06	233	2168	Tissue development
1.62E-06	81	563	Heart development
2.08E-06	335	3354	Macromolecule localization
2.33E-06	221	2046	Organonitrogen compound biosynthetic process
2.38E-06	97	724	Cardiovascular system development
2.46E-06	96	715	Vasculature development
2.46E-06	296	2905	Regulation of localization
2.61E-06	166	1444	Negative regulation of signaling
3.22E-06	424	4434	Cellular protein modification process
3.22E-06	424	4434	Protein modification process
3.42E-06	80	565	Sensory organ development
3.42E-06	165	1440	Negative regulation of cell communication
3.42E-06	74	508	Regulation of cell morphogenesis
3.86E-06	85	615	Embryonic morphogenesis
4.38E-06	312	3113	Intracellular signal transduction
4.81E-06	128	1054	Central nervous system development
4.85E-06	206	1901	Positive regulation of molecular function
5.39E-06	101	779	Brain development
5.56E-06	309	3087	Cellular localization
5.93E-06	124	1017	Negative regulation of developmental process
6.08E-06	83	603	Blood vessel morphogenesis
6.39E-06	61	396	Ossification
7.19E-06	343	3498	Positive regulation of macromolecule metabolic process
7.19E-06	87	645	Muscle structure development
7.46E-06	54	335	Regulation of developmental growth
8.77E-06	61	400	Muscle tissue development
9.48E-06	280	2768	Regulation of cellular protein metabolic process
1.01E-05	123	1018	Positive regulation of cell differentiation
1.09E-05	72	506	Regulation of neuron projection development
1.10E-05	78	564	Positive regulation of nervous system development
1.35E-05	345	3547	Response to organic substance
1.35E-05	47	280	Connective tissue development
1.38E-05	323	3287	Cell surface receptor signaling pathway
1.38E-05	64	434	Synapse organization
1.52E-05	217	2060	Positive regulation of gene expression
1.66E-05	299	3011	Organic substance transport
1.72E-05	61	409	Lipid localization
1.86E-05	50	310	Regulation of cell morphogenesis involved in differentiation
2.15E-05	44	259	Heart morphogenesis
2.53E-05	58	386	Striated muscle tissue development

2.57E-05	448	4820	Regulation of response to stimulus
2.82E-05	341	3529	Regulation of molecular function
2.88E-05	71	511	Angiogenesis
3.00E-05	222	2139	Movement of cell or subcellular component
3.00E-05	31	155	Appendage morphogenesis
3.00E-05	31	155	Limb morphogenesis
3.07E-05	10	20	Positive regulation of transforming growth factor beta production
3.69E-05	241	2364	Establishment of localization in cell
4.10E-05	189	1774	Cell-cell signaling
4.78E-05	138	1214	Ion transmembrane transport
4.86E-05	38	216	Cartilage development
5.07E-05	335	3482	Positive regulation of cellular metabolic process
5.59E-05	73	541	Skeletal system development
6.11E-05	216	2093	Protein phosphorylation
6.77E-05	147	1321	Negative regulation of signal transduction
6.79E-05	294	3006	Regulation of protein metabolic process
7.06E-05	256	2561	Response to external stimulus
7.60E-05	183	1725	Response to oxygen-containing compound
7.75E-05	34	187	Regulation of axonogenesis
8.32E-05	201	1932	Regulation of protein modification process
9.07E-05	25	117	Response to retinoic acid
9.35E-05	58	404	Muscle organ development
9.50E-05	40	240	Developmental growth involved in morphogenesis
9.55E-05	57	395	Positive regulation of cell projection organization
9.56E-05	89	712	Supramolecular fiber organization
9.56E-05	183	1733	Transmembrane transport
0.000103	19	75	Cellular response to retinoic acid
0.000118	199	1921	Locomotion
0.00014	37	218	Osteoblast differentiation
0.000141	254	2564	Negative regulation of nitrogen compound metabolic process
0.000143	105	887	Import into cell
0.000157	320	3351	Positive regulation of nitrogen compound metabolic process
0.000163	34	194	Morphogenesis of a branching epithelium
0.000164	254	2570	Phosphorylation
0.000175	62	452	Embryonic organ development
0.000177	219	2165	Cell proliferation
0.000184	54	376	Positive regulation of neuron differentiation
0.000206	25	123	Chondrocyte differentiation
0.000207	336	3556	Cellular component biogenesis
0.000242	203	1991	Regulation of transport
0.000252	206	2027	Regulation of intracellular signal transduction
0.000263	9	20	Trachea development
0.000284	52	363	In utero embryonic development
0.000284	57	411	Forebrain development
0.000287	62	460	Extracellular structure organization
0.000316	12	36	Regulation of transforming growth factor beta production
0.000321	37	227	Developmental cell growth
0.000328	177	1704	Response to endogenous stimulus
0.000373	52	367	Lipid transport
0.000397	65	495	Positive regulation of neurogenesis
0.000401	313	3309	Cellular component assembly
0.000401	32	186	Appendage development
0.000401	32	186	Limb development
0.000444	148	1386	Cytoskeleton organization
0.000464	29	162	Branching morphogenesis of an epithelial tube
0.000471	49	342	Negative regulation of cell development

0.000484	69	539	Response to extracellular stimulus
0.000489	198	1959	Intracellular transport
0.000529	28	155	Morphogenesis of embryonic epithelium
0.000529	263	2724	Negative regulation of cellular metabolic process
0.000531	91	769	Endocytosis
0.000531	54	392	Extracellular matrix organization
0.000531	12	38	Transforming growth factor beta production
0.000542	23	115	Bone morphogenesis
0.000588	43	289	Positive regulation of neuron projection development
0.000595	86	719	Tissue morphogenesis
0.000609	34	208	Morphogenesis of a branching structure
0.000616	330	3536	Cellular response to chemical stimulus
0.000624	134	1241	Cation transport
0.000639	304	3224	Negative regulation of metabolic process
0.000658	31	183	Organ growth
0.000658	59	445	Reproductive structure development
0.000658	38	245	Skeletal system morphogenesis
0.000658	243	2498	Regulation of catalytic activity
0.000658	15	58	Lung morphogenesis
0.000661	105	925	Regulation of cellular localization
0.000675	158	1514	Positive regulation of catalytic activity
0.000679	17	72	Endochondral bone morphogenesis
0.000698	39	255	Cell junction assembly
0.00073	41	274	Axon guidance
0.00073	189	1872	Regulation of phosphorus metabolic process
0.000731	283	2980	Negative regulation of macromolecule metabolic process
0.000769	50	360	Response to acid chemical
0.000772	141	1327	Carbohydrate derivative metabolic process
0.000774	41	275	Neuron projection guidance
0.000774	66	519	Regulation of GTPase activity
0.000789	59	449	Reproductive system development
0.000814	170	1658	Negative regulation of cellular biosynthetic process
0.00086	283	2988	Protein localization
0.000929	129	1199	Cellular response to oxygen-containing compound
0.000929	57	432	Glycoprotein metabolic process
0.000929	87	741	Positive regulation of cell death
0.000929	188	1870	Regulation of phosphate metabolic process
0.000962	178	1756	Regulation of cell proliferation
0.001051	29	171	Neuron projection extension
0.001051	70	566	Positive regulation of cell development
0.001126	171	1681	Negative regulation of biosynthetic process
0.001126	32	198	Actomyosin structure organization
0.001126	64	506	Response to nutrient levels
0.00115	29	172	Cardiac chamber development
0.001185	99	876	Negative regulation of transcription by RNA polymerase II
0.001195	13	48	Chondrocyte development
0.001292	27	156	Placenta development
0.001296	155	1503	Regulation of protein phosphorylation
0.001364	277	2938	Cellular response to organic substance
0.001384	81	687	Actin cytoskeleton organization
0.001413	30	183	Regulation of cell size
0.001413	155	1506	Cell migration
0.00142	52	390	Regulation of cellular component size
0.001426	35	228	Bone development
0.001477	83	710	Regulation of growth
0.001484	7	15	Pre-miRNA processing

0.001531	30	184	Regulation of cell projection assembly
0.001539	49	362	Muscle cell differentiation
0.001591	47	343	Cellular response to external stimulus
0.001591	28	167	Neural tube development
0.001625	19	93	Roof of mouth development
0.001631	148	1432	Cellular response to endogenous stimulus
0.001637	110	1006	Response to lipid
0.001637	64	514	Establishment of organelle localization
0.001664	47	344	Epithelial tube morphogenesis
0.001678	113	1040	Response to hormone
0.001687	86	746	Anterograde trans-synaptic signaling
0.001687	86	746	Chemical synaptic transmission
0.00182	85	737	Negative regulation of cell proliferation
0.001838	46	336	Regulation of cation transmembrane transport
0.001838	43	307	Cell junction organization
0.001907	42	298	Regulation of angiogenesis
0.001907	86	749	Negative regulation of cellular component organization
0.001998	48	357	Glycoprotein biosynthetic process
0.001998	18	87	Positive regulation of axonogenesis
0.002011	168	1670	Cell motility
0.002011	168	1670	Localization of cell
0.002019	45	328	Regulation of vasculature development
0.002103	26	153	Tube formation
0.002198	98	883	Membrane organization
0.002204	246	2592	Nitrogen compound transport
0.002282	100	906	Cation transmembrane transport
0.002347	75	637	Anion transport
0.002356	166	1653	Regulation of phosphorylation
0.002396	74	627	Behavior
0.002409	33	217	Cardiac muscle tissue development
0.002434	25	146	Regulation of lipid localization
0.002463	86	756	Trans-synaptic signaling
0.002562	69	576	Positive regulation of locomotion
0.002662	29	182	Regulation of plasma membrane bounded cell projection assembly
0.002705	83	726	Organelle localization
0.002777	175	1764	Negative regulation of response to stimulus
0.002927	65	537	Negative regulation of intracellular signal transduction
0.002947	108	1002	Regulation of cellular component biogenesis
0.002947	42	305	Embryonic organ morphogenesis
0.00307	16	75	Lens development in camera-type eye
0.003112	86	762	Synaptic signaling
0.003131	30	193	Regulation of ossification
0.003218	17	83	Lamellipodium organization
0.003225	54	426	Actin filament organization
0.003225	87	774	Negative regulation of cell differentiation
0.003257	20	107	Negative regulation of developmental growth
0.003287	160	1597	Negative regulation of macromolecule biosynthetic process
0.003372	204	2113	Positive regulation of biosynthetic process
0.003372	12	47	Embryonic cranial skeleton morphogenesis
0.003372	12	47	Cartilage development involved in endochondral bone morphogenesis
0.00351	55	438	Muscle system process
0.003517	21	116	Bone mineralization
0.003594	30	195	Gastrulation
0.003594	48	368	Eye development
0.003594	47	358	Cell-substrate adhesion
0.003594	34	232	Ear development

0.003594	82	723	Response to growth factor
0.003603	36	251	Neuron apoptotic process
0.003603	19	100	Cell-substrate junction assembly
0.003687	65	543	Wnt signaling pathway
0.003798	109	1022	Positive regulation of cell proliferation
0.003879	23	134	Lipoprotein metabolic process
0.003896	367	4098	Organelle organization
0.00401	65	545	Cell-cell signaling by wnt
0.00401	19	101	Triglyceride metabolic process
0.00401	189	1945	Positive regulation of signaling
0.00401	194	2004	Homeostatic process
0.004028	38	272	Cellular response to extracellular stimulus
0.004237	23	135	Epithelial to mesenchymal transition
0.004246	6	13	Negative regulation of macrophage derived foam cell differentiation
0.004246	212	2220	Vesicle-mediated transport
0.004284	56	453	Negative regulation of phosphorylation
0.004325	188	1937	Positive regulation of cell communication
0.004325	31	207	Sulfur compound biosynthetic process
0.004325	20	110	Regulation of extent of cell growth
0.00434	48	372	Visual system development
0.004359	166	1680	Positive regulation of cellular protein metabolic process
0.004359	66	558	Positive regulation of cellular component movement
0.004388	75	654	Cell surface receptor signaling pathway involved in cell-cell signaling
0.004456	63	527	Regulation of anatomical structure size
0.004528	60	496	Organic anion transport
0.004538	46	353	Muscle contraction
0.004538	13	56	Sulfur compound catabolic process
0.004558	219	2308	Establishment of protein localization
0.004561	12	49	Negative regulation of lipid localization
0.004574	152	1520	Lipid metabolic process
0.004574	40	294	Negative regulation of neurogenesis
0.004681	42	314	Negative regulation of nervous system development
0.004756	32	218	Cellular response to acid chemical
0.004771	104	975	Response to organic cyclic compound
0.004771	85	765	Regulation of cell adhesion
0.004852	151	1511	Negative regulation of cellular macromolecule biosynthetic process
0.004882	18	95	Regulation of axon extension
0.004893	35	247	Dendrite development
0.004893	73	636	Negative regulation of protein modification process
0.004893	42	315	Vesicle localization
0.004903	215	2265	Amide transport
0.004978	81	723	Regulation of ion transport
0.005039	77	680	Positive regulation of apoptotic process
0.005058	88	800	Carbohydrate derivative biosynthetic process
0.005085	9	30	Chondrocyte differentiation involved in endochondral bone morphogenesis
0.005089	22	129	Cardiac chamber morphogenesis
0.005163	11	43	Endochondral bone growth
0.005192	86	779	Actin filament-based process
0.005237	23	138	Epithelial tube formation
0.005285	36	258	Cell cycle arrest
0.005325	34	239	Cell-matrix adhesion
0.005325	21	121	Axon extension
0.005378	25	156	Localization within membrane
0.00552	22	130	Acylglycerol metabolic process
0.00552	22	130	Embryonic limb morphogenesis

0.00552	22	130	Embryonic appendage morphogenesis
0.005532	64	544	Positive regulation of cell motility
0.005532	14	65	Positive regulation of neuron apoptotic process
0.005532	40	298	Establishment of vesicle localization
0.005532	78	694	Cellular response to growth factor stimulus
0.005538	199	2082	Positive regulation of cellular biosynthetic process
0.005711	178	1835	Regulation of cell death
0.005713	10	37	Regulation of voltage-gated calcium channel activity
0.005851	55	451	Pattern specification process
0.005901	211	2228	Peptide transport
0.005901	42	319	Camera-type eye development
0.005965	22	131	Neutral lipid metabolic process
0.006011	12	51	Heterophilic cell-cell adhesion via plasma membrane cell adhesion molecules
0.006011	60	504	Cell growth
0.006056	77	686	Positive regulation of programmed cell death
0.006154	81	730	Transmembrane receptor protein tyrosine kinase signaling pathway
0.006157	9	31	Substrate-dependent cell migration
0.006561	139	1386	Epithelium development
0.006573	58	485	Regulation of ion transmembrane transport
0.006625	17	90	Embryonic placenta development
0.006723	26	168	Actin filament bundle organization
0.006731	30	205	Inner ear development
0.006824	56	465	Second-messenger-mediated signaling
0.006876	69	603	Regulation of system process
0.006903	21	124	Gland morphogenesis
0.007008	48	383	Sensory system development
0.007008	90	833	Positive regulation of hydrolase activity
0.007126	14	67	Regulation of postsynaptic membrane neurotransmitter receptor levels
0.007126	27	178	Muscle cell development
0.007149	18	99	Negative regulation of cell morphogenesis involved in differentiation
0.007164	43	333	Protein localization to cell periphery
0.007164	3	3	Negative regulation of follicle-stimulating hormone secretion
0.007164	135	1344	Positive regulation of cellular component organization
0.007164	3	3	Foregut regionalization
0.007164	3	3	Lung field specification
0.007164	3	3	Lung induction
0.007164	3	3	Cardiac muscle thin filament assembly
0.007275	56	467	Modulation of chemical synaptic transmission
0.007277	189	1978	Positive regulation of macromolecule biosynthetic process
0.007277	140	1403	Regulation of hydrolase activity
0.007301	173	1789	Positive regulation of RNA metabolic process
0.007334	67	584	Plasma membrane bounded cell projection assembly
0.007357	5	10	Epithelial cell proliferation involved in lung morphogenesis
0.007511	56	468	Regulation of trans-synaptic signaling
0.007511	111	1072	Enzyme linked receptor protein signaling pathway
0.007511	429	4923	Regulation of gene expression
0.007526	135	1347	Regulation of organelle organization
0.007587	187	1957	Cell cycle
0.007587	25	161	Glycosaminoglycan metabolic process
0.007587	24	152	Regulation of epithelial cell differentiation
0.007616	189	1981	Cellular protein localization
0.007691	8	26	Central nervous system projection neuron axonogenesis
0.007824	14	68	Cranial skeletal system development
0.007884	68	597	Cell projection assembly
0.007981	11	46	Bone growth

0.007981	26	171	Aminoglycan metabolic process
0.007981	44	346	Urogenital system development
0.007981	105	1007	MAPK cascade
0.007981	106	1018	Signal transduction by protein phosphorylation
0.007981	19	109	Regulation of lipid transport
0.007981	6	15	Positive regulation of keratinocyte differentiation
0.007981	22	135	Regulation of embryonic development
0.00814	65	566	Regulation of transmembrane transport
0.00814	119	1167	Cellular lipid metabolic process
0.008208	28	190	Cytoskeleton-dependent intracellular transport
0.00821	173	1796	Positive regulation of protein metabolic process
0.00828	45	357	Regulation of small GTPase mediated signal transduction
0.008533	29	200	Synaptic vesicle cycle
0.008533	21	127	Negative regulation of epithelial cell proliferation
0.008533	143	1446	Negative regulation of RNA metabolic process
0.008571	34	248	Regulation of synapse structure or activity
0.008766	156	1599	Positive regulation of transcription, DNA-templated
0.008812	9	33	Specification of animal organ identity
0.009145	130	1298	Regulation of cell cycle
0.00919	26	173	Regulation of cation channel activity
0.009316	25	164	Actin filament bundle assembly
0.009316	25	164	Striated muscle cell development
0.009417	52	432	Regulation of cell growth
0.009417	66	581	Morphogenesis of an epithelium
0.009417	33	240	Cellular response to nutrient levels
0.009417	8	27	Cardiac myofibril assembly
0.009417	8	27	Negative regulation of cartilage development
0.009417	189	1993	Cellular macromolecule localization
0.009417	7	21	Hematopoietic stem cell proliferation
0.00943	13	62	Animal organ formation
0.009458	19	111	Glycosaminoglycan biosynthetic process
0.009458	76	690	Negative regulation of cell cycle
0.009643	194	2054	Negative regulation of gene expression
0.009645	56	475	Phospholipid metabolic process
0.009693	36	270	Sensory organ morphogenesis
0.009891	205	2187	Protein transport
0.009891	30	212	Mesenchymal cell differentiation
0.009937	24	156	Establishment of synaptic vesicle localization
0.009937	24	156	Synaptic vesicle transport
0.009962	108	1050	Regulation of catabolic process

Table S3. Cross design used to produce RNA sequencing libraries for F1 offspring sampled at 2 days post fertilization (dpf), 8 dpf, and 20 dpf. CP = Crescent Pond, OL = Osprey Lake, and LL = Little Lake.

Mother	Father	Stage	Libraries	F1
CP molluscivore	CP scale-eater	2 dpf	3	hybrid
OL scale-eater	OL molluscivore	2 dpf	3	hybrid
CP molluscivore	CP scale-eater	8 dpf	3	hybrid
OL scale-eater	OL molluscivore	8 dpf	3	hybrid
CP molluscivore	CP molluscivore	2 dpf	3	purebred
CP scale-eater	CP scale-eater	2 dpf	3	purebred
OL molluscivore	OL molluscivore	2 dpf	3	purebred
OL scale-eater	OL scale-eater	2 dpf	3	purebred
CP molluscivore	CP molluscivore	8 dpf	3	purebred
CP scale-eater	CP scale-eater	8 dpf	5	purebred
OL molluscivore	OL molluscivore	8 dpf	5	purebred
OL scale-eater	OL scale-eater	8 dpf	5	purebred
CP molluscivore	CP molluscivore	20 dpf	3	purebred
CP scale-eater	CP scale-eater	20 dpf	2	purebred
LL molluscivore	LL molluscivore	20 dpf	3	purebred

Table S4. 384 genes differentially expressed during at least one developmental stage and within 20 kb of highly differentiated SNPs (*Fst* > 0.72 (genome-wide 99th percentile) and *Dxy* > 0.0083 (genome-wide 90th percentile)).

abca1	*col14a1*	*gbgt1*	*map2k6*	*pnpla2*	*sowaha*
abcb1	*coq7*	*gdf10*	*map3k20*	*pon2*	*sowahc*
abcd4	*coro1c*	*gga1*	*map6*	*ppa2*	*sox17a*
acss1	*cox6b1*	*ggct*	*mark1*	*ppara*	*spice1*
acy3.2	*cpa2*	*gimap2*	*mcl1*	*ppfibp1*	*spns2*
adamts10	*crabp2*	*gimap4*	*mecom*	*ppp1r14b*	*sprn*
adgrf5	*crot*	*gimap5*	*med1*	*ppp1r3a*	*srpk2*
afg3l1	*crybb1*	*gimap7*	*mefv*	*ppp2r3b*	*srpra*
ago1	*csad*	*gimap8*	*meltf*	*prc1*	*st3gal1*
ago2	*csf1r1*	*gimap9*	*mettl14*	*prdx3*	*stac3*
agps	*csga*	*gjc1*	*mfap4*	*prelid3a*	*stag2*
alox15b	*cspg5*	*glb1l2*	*mllt10*	*prps2*	*steap4*
alpi	*csrp2*	*glcci1*	*mmp13*	*prr16*	*stmn2*
ampd1	*ctbp1*	*glipr2*	*mob3a*	*psap*	*strip1*
ank1	*ctdsp1*	*golga4*	*mov10b.1*	*psbp1*	*syndig1l*
ap3m1	*ctsk*	*gpm6a*	*mpdu1*	*ptger4*	*synpcc7002_a1628*
apoa4	*cyp27b1*	*gpm6b*	*mpdz*	*ptx2*	*tacc2*
apoh	*cyp2k1*	*gpr155*	*mpp3*	*pwp1*	*tagln3*
arel1	*cyr61*	*gpr21*	*mpst*	*pxk*	*tanc2*
arfgef1	*d7ertd443e*	*gprc6a*	*mpv17l*	*pycr3*	*tbrg4*
arhgap24	*ddit3*	*grin3a*	*mr1*	*rab38*	*tbx5a*
arl6	*ddx49*	*grina*	*mrc1*	*ralyl*	*tc2n*
arsi	*dennd3*	*grpel1*	*mrc2*	*rasip1*	*tcap*
art5	*dgkq*	*grwd1*	*mtap*	*rdh12*	*tead3*
asph	*dixdc1a*	*gvin1*	*myadm*	*reep1*	*tecta*
atad2	*dlx6a*	*gzmb*	*mylipa*	*rnaseh2c*	*tgm5*
atad2	*dnajc22*	*h2-eb1*	*myoz2*	*rnf182*	*thbs1*
atf4	*dph5*	*h2-q10*	*nacad*	*rnf214*	*tjp3*
aurkaip1	*dpp3*	*hbs1l*	*nachra9*	*rnf24*	*tmed2*
b3galt2	*dpysl3*	*heg*	*ncoa2*	*rpl19*	*tmem130*
b3gnt3	*dsp*	*henmt1*	*ncoa3*	*rpl4-a*	*tmem179*
bcl7a	*dst*	*hephl1*	*nefm*	*rps14*	*tmem67*
best2	*dupd1*	*herpud1*	*ngdn*	*rtkn2*	*tnc*
bmb	*dync2li1*	*hes5*	*nipblb*	*rtn2*	*tox4*
bscl2	*e2f2*	*hhatl*	*nmd3*	*rxylt1*	*tpi1b*
c8orf76	*eef1d*	*hint1*	*nmt2*	*sag*	*trib2*
ca14	*efna2*	*hnf1a*	*noct*	*sall2*	*trim21*
cald1	*ehbp1l1*	*hnrnpc*	*nol7*	*samd8*	*tsc2*
capn9	*ehhadh*	*hnrnpl*	*nsmce2*	*sbk1*	*tspan33*
card8	*eif2ak1*	*homer1*	*nsmce4a*	*sdha*	*tsr3*
casq1	*emilin2*	*hspg2*	*nthl1*	*sel1l3*	*ttc7a*
cavin4a	*endod1*	*hus1*	*nudt6*	*sept8a*	*ubxn1*
cbfa2t2	*endouc*	*ian9*	*nwd1*	*serpinb1*	*ugp2*
cbln1	*ermap*	*igfbp6*	*nxpe3*	*serpinc1*	*umps*
ccdc86	*erp44*	*il6st*	*nxpe3*	*serpinh1*	*urad*
ccomt	*exosc4*	*ildr1*	*ogn*	*sesn1*	*urgcp*
cd226	*eya2*	*ino80c*	*pabpn1*	*sh3bgr*	*usp2*
cd63	*f2r*	*ints6*	*paox*	*shroom3*	*usp6nl*
cd93	*fabp3*	*invs*	*papln*	*sik2*	*vash1*
cdc25a	*fabp4*	*ism1*	*parp9*	*slc12a3*	*vim*
cdh17	*fabp7*	*itga10*	*pcmtd1*	*slc14a2*	*vstm2a*

cdhr2	*fam102b*	*itgb3*	*pdhb*	*slc16a1*	*vsx2*
cdk5r1	*fam118b*	*itpk1*	*pdk2*	*slc1a4*	*vwc2l*
cetp	*fbln1*	*jak1*	*phlda2*	*slc22a5*	*vwde*
cfh	*fbxo45*	*jund*	*pim2*	*slc24a2*	*wasf3*
cgn	*fgfr3*	*kera*	*pkd1l2*	*slc2a9*	*washc2*
chia	*flt3*	*kif20a*	*pkia*	*slc43a3*	*washc5*
chrna7	*fndc7*	*kif2c*	*pkp1*	*slc45a4*	*wdfy2*
cideb	*foxj2*	*klf5*	*pla2g12b*	*slc46a3*	*wnt9b*
cmbl	*fsd1*	*l-2*	*pla2g16*	*slc6a13*	*yjefn3*
cngb3	*fuca1*	*lim2*	*plb1*	*slc7a2*	*zfhx4*
cnpy3	*fuom*	*lox*	*pls3*	*slc7a8*	*zg16*
cnrip1	*gab1*	*mag*	*plscr2*	*smarcd2*	*zgc:112255*
col10a1	*gba3*	*maip1*	*pno1*	*snrpd1*	*znf214*

Table S5. 384 genes (Table S4) differentially expressed during at least one developmental stage and within 20 kb of highly differentiated SNPs (*Fst* > 0.72 (genome-wide 99th percentile) and *Dxy* > 0.0083 (genome-wide 90th percentile)) were enriched for 87 biological processes.

Enrichment FDR	Genes in list	Total genes	Functional Category
0.006892	25	637	Anion transport
0.006892	20	409	Lipid localization
0.006892	22	496	Organic anion transport
0.006892	101	4459	Cell differentiation
0.006892	14	232	Fat cell differentiation
0.006892	104	4671	Cellular developmental process
0.011766	4	13	Negative regulation of macrophage derived foam cell differentiation
0.014613	10	146	Regulation of lipid localization
0.014613	17	367	Lipid transport
0.014613	5	30	Regulation of macrophage derived foam cell differentiation
0.014613	11	171	Monocarboxylic acid transport
0.014613	12	207	Sulfur compound biosynthetic process
0.014613	4	15	Positive regulation of keratinocyte differentiation
0.014613	32	1017	Negative regulation of developmental process
0.014613	95	4319	Regulation of biological quality
0.016409	35	1167	Cellular lipid metabolic process
0.017319	9	131	Neutral lipid metabolic process
0.017319	9	130	Acylglycerol metabolic process
0.017319	8	101	Triglyceride metabolic process
0.017319	80	3570	Phosphate-containing compound metabolic process
0.017319	47	1775	Ion transport
0.017319	8	101	Fatty acid transport
0.017319	17	401	Regulation of lipid metabolic process
0.017319	5	33	Triglyceride catabolic process
0.017319	28	860	Tube morphogenesis
0.017319	3	8	Platelet dense granule organization
0.017649	5	36	Foam cell differentiation
0.017649	5	36	Macrophage derived foam cell differentiation
0.018775	24	712	Supramolecular fiber organization
0.018775	41	1520	Lipid metabolic process
0.018775	80	3597	Phosphorus metabolic process
0.018775	15	343	Organic acid transport
0.018775	8	109	Regulation of lipid transport
0.018775	18	460	Extracellular structure organization
0.018775	15	343	Carboxylic acid transport
0.021627	8	112	Negative regulation of angiogenesis
0.023719	8	114	Negative regulation of blood vessel morphogenesis
0.023719	23	687	Blood vessel development
0.023719	6	63	Regulation of epidermal cell differentiation
0.023719	4	23	Positive regulation of epidermal cell differentiation
0.023829	31	1062	Tube development
0.023829	4	24	Reverse cholesterol transport
0.023829	4	24	Intermediate filament organization
0.023829	5	42	Regulation of keratinocyte differentiation
0.023829	21	603	Blood vessel morphogenesis
0.024444	6	65	Aminoglycan catabolic process
0.026837	4	25	Negative regulation of lipid storage

0.027035	15	366	Lipid catabolic process
0.028655	2	3	Negative regulation of protein deubiquitination
0.028655	8	125	Negative regulation of vasculature development
0.028655	23	715	Vasculature development
0.028655	3	12	Pigment accumulation
0.028655	3	12	Cellular pigment accumulation
0.028655	5	46	Neutral lipid catabolic process
0.028655	5	46	Acylglycerol catabolic process
0.028655	63	2763	Regulation of developmental process
0.028655	35	1286	Negative regulation of multicellular organismal process
0.028655	3	12	Phospholipid homeostasis
0.028655	3	12	Positive regulation of phospholipid biosynthetic process
0.031105	33	1199	Negative regulation of protein metabolic process
0.031105	23	724	Cardiovascular system development
0.031656	14	342	Negative regulation of cell development
0.031656	4	28	Keratan sulfate biosynthetic process
0.033233	5	49	Negative regulation of lipid localization
0.033433	3	13	Regulation of integrin activation
0.033754	18	511	Angiogenesis
0.034326	5	50	Positive regulation of phospholipid metabolic process
0.034326	32	1164	Anatomical structure formation involved in morphogenesis
0.035495	49	2046	Organonitrogen compound biosynthetic process
0.035647	6	76	Lipid storage
0.035647	15	392	Extracellular matrix organization
0.035647	31	1123	Negative regulation of cellular protein metabolic process
0.035647	5	51	Intermediate filament cytoskeleton organization
0.037242	62	2768	Regulation of cellular protein metabolic process
0.037242	5	52	Intermediate filament-based process
0.039705	5	53	Regulation of lipid storage
0.039705	61	2724	Negative regulation of cellular metabolic process
0.041139	72	3354	Macromolecule localization
0.041139	2	4	Negative regulation of lipoprotein metabolic process
0.041139	58	2564	Negative regulation of nitrogen compound metabolic process
0.041139	66	3011	Organic substance transport
0.04122	3	15	Negative regulation of substrate adhesion-dependent cell spreading
0.04275	8	141	Regulation of JAK-STAT cascade
0.04414	4	33	Keratan sulfate metabolic process
0.045431	5	56	Zymogen activation
0.045431	5	56	Sulfur compound catabolic process
0.0487	8	145	Regulation of STAT cascade

Table S6. Proportion of transcribed genes containing sites that were alternatively homozygous between parents and heterozygous in their F1 (Mom = A/A, Dad = a/a, F1 hybrid = A/a).

lake	stage	genes expressed	heterozygous genes	% heterozygous
Crescent Pond	8 dpf	13451	3254	0.241915
Osprey Lake	8 dpf	13451	2488	0.184968
Crescent Pond	2 dpf	12974	3697	0.284955
Osprey Lake	2 dpf	12974	3240	0.24973

Table S7. 37 genes differentially expressed during at least one developmental stage, within 20 kb of highly differentiated SNPs (*Fst* > 0.72 (genome-wide 99th percentile) and *Dxy* > 0.0083 (genome-wide 90th percentile)), showing *cis*-regulatory divergence between species.

abcd4
acy3.2
apoh
atad2
cideb
cnpy3
cox6b1
csad
dpp3
dync2li1
ehhadh
emilin2
exosc4
flt3
fuom
gimap4
gpm6a
grina
hspg2
ints6
kif20a
mecom
mpst
myadm
nsmce4a
nxpe3
pycr3
rasip1
rdh12
rnaseh2c
slc1a4
slc7a8
stag2
tead3
tnc
tox4
usp6nl

Table S8. Predicted transcription factor binding sites (JASPAR database) altered by genetic variants fixed between species.

gene region	allele	transcription factor	matrix ID	binding sequence	relative profile score
dync2li1	reference	NFIC	MA0161.1	TTGGCA	1.00000
dync2li1	reference	NFIA	MA0670.1	AATGCCAAGT	0.98703
dync2li1	reference	NFIX	MA0671.1	AATGCCAAG	0.98551
dync2li1	reference	ZNF384	MA1125.1	TCAGAAAAAAAA	0.96526
dync2li1	reference	HOXA5	MA0158.1	CTGTAATT	0.96148
dync2li1	reference	Gata1	MA0035.1	TGATGC	0.95591
dync2li1	reference	MYB	MA0100.3	CACAACTGGC	0.95232
dync2li1	reference	Prrx2	MA0075.1	AATTA	1.00000
dync2li1	reference	Stat5a	MA1624.1	GTTCCAAGAATT	0.98454
dync2li1	alternate	Prrx2	MA0075.1	AATTA	1.00000
dync2li1	alternate	Stat5a	MA1624.1	GTTCCAAGAATT	0.98454
pycr	reference	GATA2	MA0036.1	AGATA	0.97565
pycr	reference	MZF1	MA0056.1	GGGGGA	0.96199
pycr	alternate	PLAGL2	MA1548.1	TGGGCCCCCA	0.98454
pycr	alternate	GATA2	MA0036.1	AGATA	0.97565

Table S9. Allele-specific expression (ASE) analyses do not disproportionally exclude genes involved in skeletal development. Genes used to measure ASE containted heterozygous sites in F1 Hybrids. Fisher's exact tests for each hybrid cross tested the null hypothesis that the proportion of heterozygous genes within an ontology was equal to the proportion of non-informative genes within an ontology. In the only two comparisons that did show a significant difference ($P < 0.05$), heterozygous genes were enriched for skeletal genes.

lake	stage	informative genes	% informative genes in cranial GO	% non-informative genes in cranial GO	% informative genes in skeletal GO	% non-informative genes in skeletal GO	*P* cranial	*P* skeletal
Crescent Pond	2 dpf	3526	0.62	0.42	0.88	0.73	0.25	0.435
Crescent Pond	8 dpf	3122	0.74	0.4	0.99	0.71	0.154	0.13
Osprey Lake	2 dpf	3115	0.51	0.46	0.9	0.73	0.767	0.354
Osprey Lake	8 dpf	2409	0.71	0.43	1.29	0.66	0.073	0.003

Fig. S1. UpSet plot (Conway et al. 2017) showing intersection across five sets: genes differentially expressed at each of the three stages (DESeq2 $P < 0.01$), genes within 20 kb of highly differentiated SNPs showing *Fst* > 0.72 (genome-wide 99th percentile), and genes within 20 kb windows showing *Dxy* > 0.0083 (genome-wide 90th percentile).

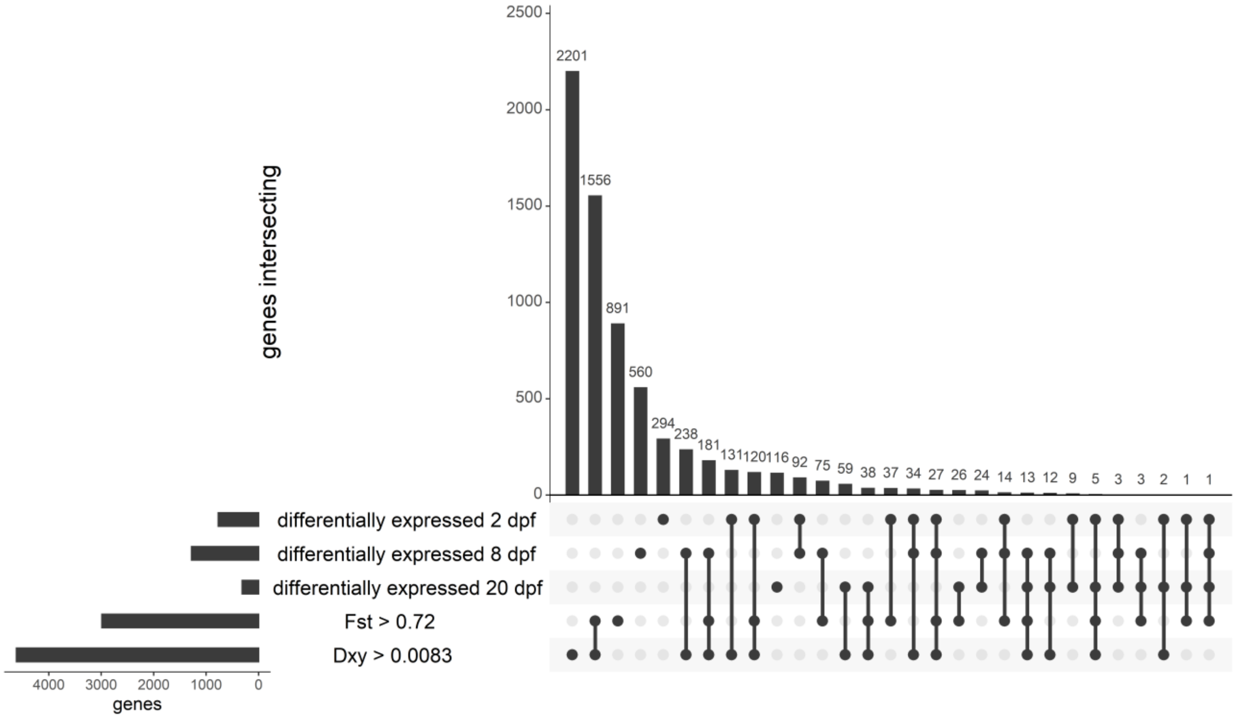

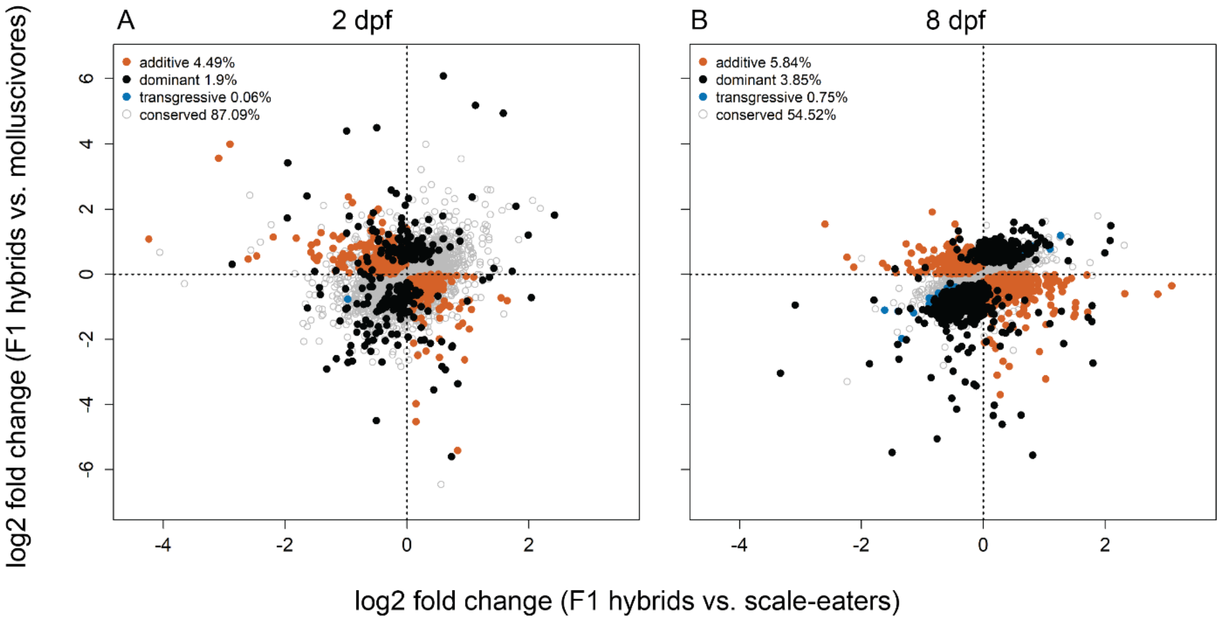

Fig. S2. We grouped species and F1 hybrids by lake population and compared expression in F1 hybrids to expression in purebred offspring to determine whether genes showed additive (red), dominant (black), or transgressive (blue) patterns of inheritance in hybrids. F1 hybrid inheritance was considered additive if hybrid gene expression was intermediate between parental populations and significantly different between parental populations. Inheritance was dominant if hybrid expression was significantly different from one parental population but not the other. Genes showing misexpression in hybrids showed transgressive inheritance, meaning hybrid gene expression was significantly higher (overdominant) or lower (underdominant) than both parental species. Panels show inheritance patterns for F1 hybrids collected at A) 2 days post fertilization and B) 8 dpf.

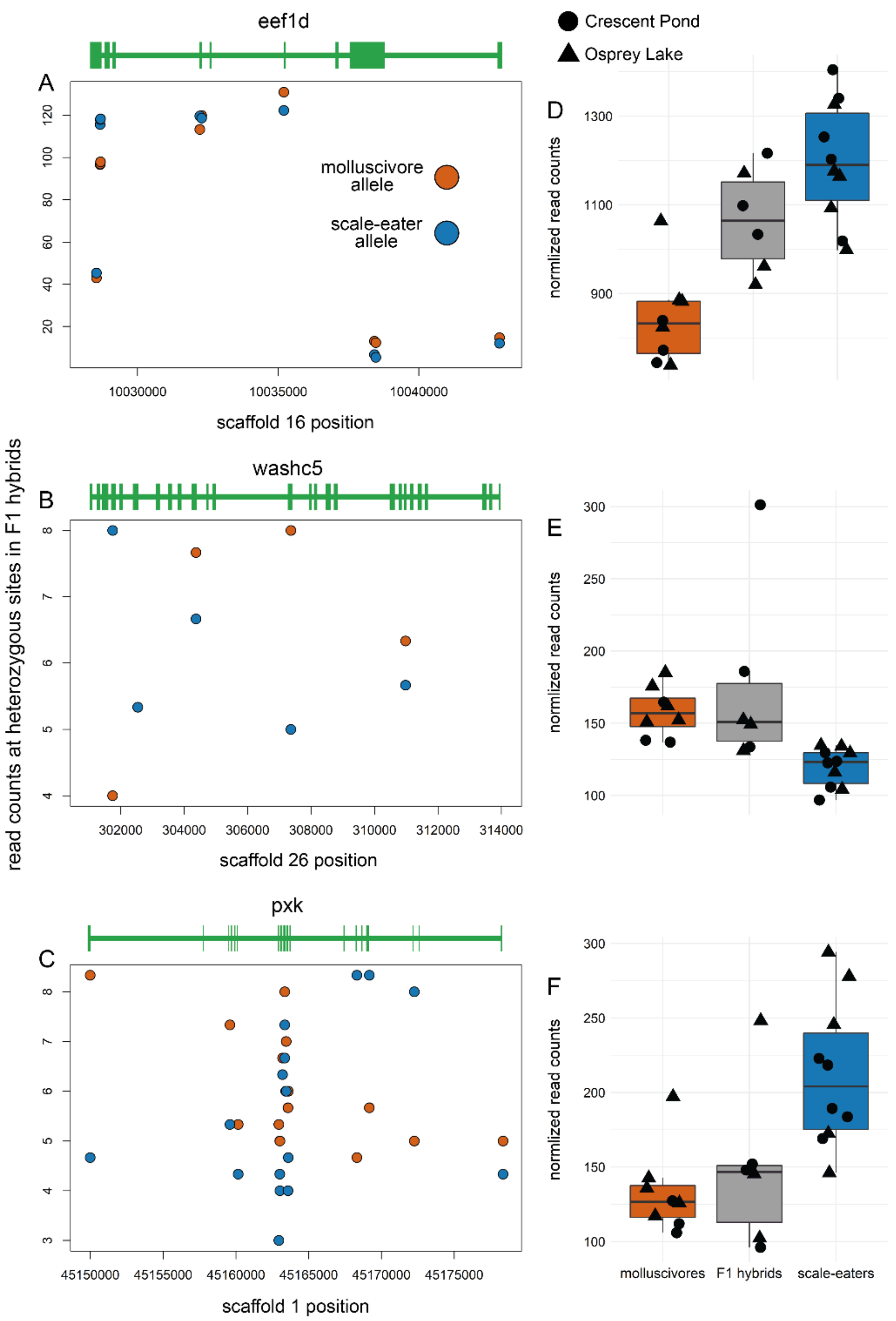

Fig. S3. Three genes near fixed variants show *trans*-regulatory divergence between trophic specialists. A-D) Mean counts for reads spanning A) *eef1d*, B) *washc5*, and C) *pxk* that match parental alleles at heterozygous sites are shown for crosses between Crescent Pond molluscivores (red) and scale-eaters (blue) at 8 dpf. D-F) Library size normalized read counts for F1 offspring from Crescent Pond (circles) and Osprey Lake (triangles) crosses. All three genes are differentially expressed between molluscivores (red) and scale-eaters (blue) at 8 dpf. Comparing the ratio of maternal and paternal allelic expression in F1 hybrids with the ratio of molluscivore and scale-eater gene expression in purebred F1 offspring indicated a significant *trans*-contribution to expression divergence.

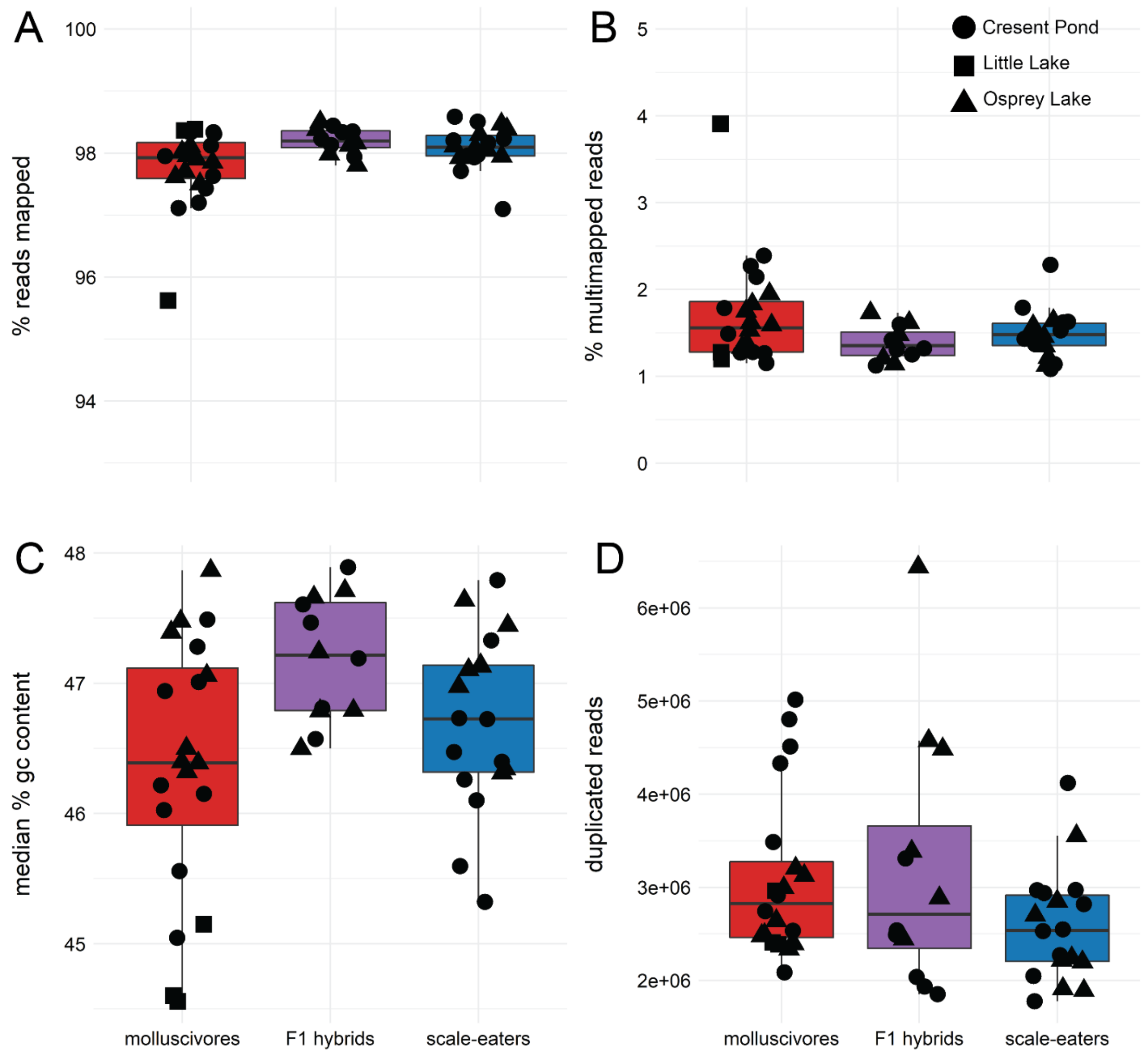

Fig. S4. Quality control measures for 50 RNAseq libraries. We did not find a difference between scale-eaters and mollusciores in A) the proportion of reads uniquely mapped to the molluscivore reference genome (Student's t-test, $P = 0.061$), B) the proportion of multimapped reads (Student's t-test, $P = 0.14$) ,C) the median GC content of aligned reads (Student's t-test, $P = 0.22$), or D) the number of duplicate reads (Student's t-test, $P = 0.05$).

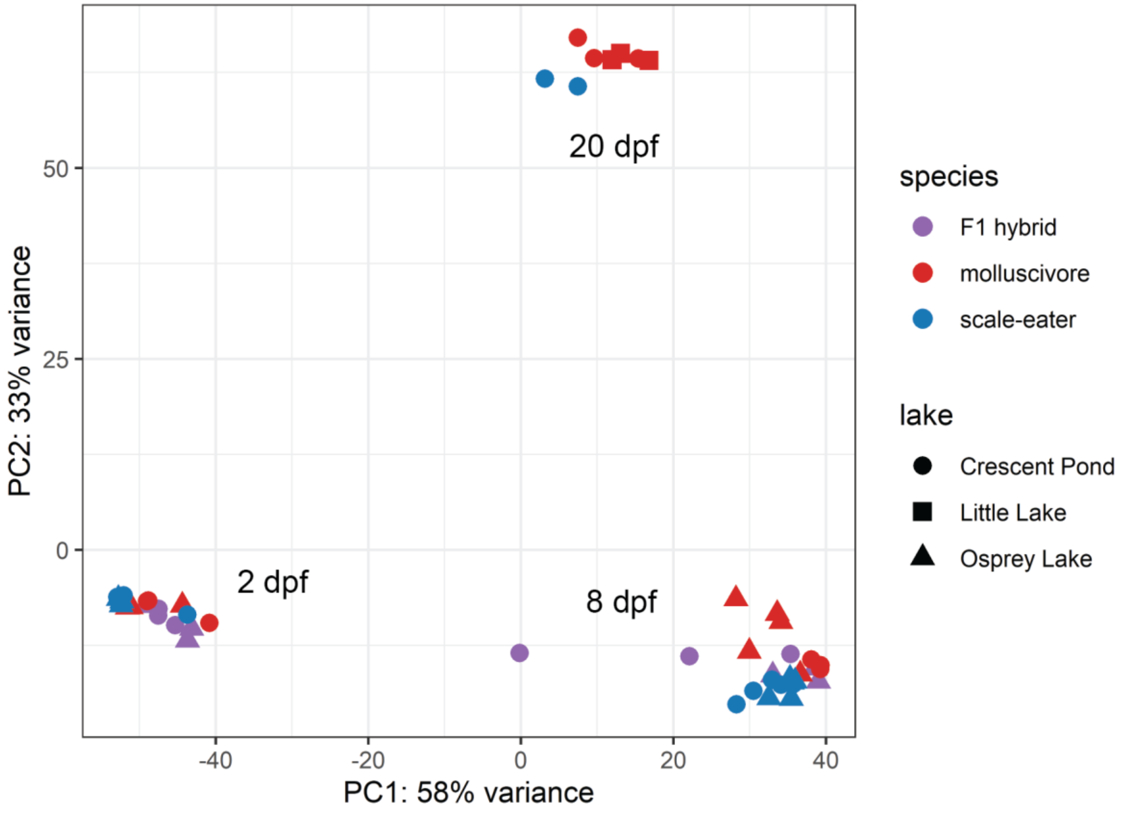

Fig. S5. Principal component analysis for 50 transcriptomes showing first two axes accounting for a combined 91% of the total variation in read counts normalized for library size.
